# 50 Rise of the (Learning) Machines: Artificial Intelligence for the Assessment of Adult Thermal Burns

**DOI:** 10.1093/jbcr/irac012.053

**Published:** 2022-03-23

**Authors:** Jeffrey E Carter, Herb A Phelan, William L Hickerson, J Michael DiMaio, Jeffrey W Shupp, James H Holmes

**Affiliations:** University Medical Center- New Orleans, New Orleans, Louisiana; LSUHSC-New Orleans Department of Surgery, New Orleans, Louisiana; Spectral MD, Avita Medical, AccessPro Med, Memphis, Tennessee; Baylor Scott and White, Dallas, Texas; MedStar Washington Hospital Center, Washington DC, District of Columbia; Atrium Health Wake Forest Baptist, Winston-Salem, North Carolina

## Abstract

**Introduction:**

Burn depth assessment (BDA) is an essential component of the physical exam used in the treatment and triage of burn injured patients. And while many specialties incorporate labs and imaging to determine diagnoses, burn professionals must rely on a physical exam that is accurate in only 70-80% of cases. Our goal was to assess the accuracy of a new imaging technology called Multispectral imaging (MSI) combined with a machine learning algorithm to aid in rapid BDA. We present the results of the first multi-center study using this technology in adult burn injuries.

**Methods:**

In a multi-center IRB-approved study, an MSI device was used to image subjects >18 years of age with thermal burn injuries. The imaging device captured a set of images measuring the reflectance of visible and near-IR light. Subjects were enrolled and imaged within 72 hours of injury with serial imaging as permitted. The images were used to develop a type of machine learning algorithm called a convolutional neural network (CNN) that could identify the regions of non-healing burn within an image. Non-healing burn areas were determined by a panel of three burn surgeons using two standards: a) images confirming 21-day spontaneous healing; or b) pathology reports detailing histologic changes from multiple punch biopsies taken prior to burn excision. From this data, an ensemble of eight separate CNN algorithms was used to automatically identify non-healing burn tissue. Training and test accuracies of the ensemble CNN were calculated using cross-validation at the level of the subject.

**Results:**

One hundred (100) adults were enrolled and imaged. The population had a mean age 45.6 ± 16.7; mean TBSA 13.0 ± 9.3; and was 31% female. From these adults, 210 burn regions were serially imaged. The estimated performance result from the ensemble CNN for identification of non-healing burn regions was AUC of 0.96. Based on the ROC curve, an ideal threshold showed an accuracy of 92.0%, sensitivity 91.9%, and specificity 92.0%.

**Conclusions:**

Our study demonstrates a non-invasive technology that rapidly determines an accurate DBA relative to traditional bedside exam. More accurate burn wound assessment could lead to avoiding unnecessary surgeries or delays in treatment and dramatic cost savings. Use of such a device in a disaster has additional value to better align a patient’s burn care needs and available resources.

